# Face-to-face, online, or blended: which method is more effective in teaching electrocardiogram to medical students

**DOI:** 10.1186/s12909-023-04546-0

**Published:** 2023-08-09

**Authors:** Aida Bazrgar, Mahdi Rahmanian, Arshin Ghaedi, Ali Heidari, Mehdi Bazrafshan, Mitra Amini, Hanieh Bazrafshan, Mahsa Ahmadpour, Hamed Bazrafshan drissi

**Affiliations:** 1 Student research committee, Shiraz University of medical science, Shiraz, Iran; 2 Cardiovascular research center, Shiraz University of medical science, Shiraz, Iran; 3 Student research committee, School of Medicine, Shiraz University of medical science, Shiraz, Iran; 4 Clinical Education Research Center, Shiraz University of Medical Sciences, Shiraz, Iran; 5 Clinical Neurology Research Center, Shiraz University of Medical Science, Shiraz, Iran

**Keywords:** Distance education, Learning, Medical students, Electrocardiography

## Abstract

**Background:**

Electrocardiogram (ECG) remains an important medical diagnostic and screening tool. This study aimed to compare the effectiveness of online classes instead of traditional face-to-face or blended methods in medical students’ ECG learning.

**Methods:**

Two hundred and fifteen medical students (including 105 (48.8%) males and 110 (51.2%) females) were studied from February 2021 to February 2022. Regardless of their grade, participants were divided into three groups: online, face-to-face, and blended. Then all participants sat for an ECG interpretation exam, and their results were compared.

**Results:**

Twenty-six (12.1%) participants were residents, and 189 (87.9%) were interns. Thirty-five (16.3%), 85 (39.5%), and 95 (44.2%) participants were taught ECG through face-to-face, online, and blended methods, respectively. Regarding participants’ preferences on teaching methods, 118 (54.9%) preferred face-to-face learning, and the remaining 97 (45.1%) chose online learning (p < 0.001). The blended method seemed more promising in almost half of the exam questions regarding teaching method effectiveness. The mean total exam score was also significantly higher in participants who were taught blended than in the others (7.20 ± 1.89, p = 0.017). Face-to-face (5.97 ± 2.33) and online teaching methods (6.07 ± 2.07) had similar efficacy according to the mean total score (p = 0.819).

**Conclusion:**

While most students preferred face-to-face learning to online learning, a blended method seemed more promising regarding students’ skill enhancement to interpret ECG.

## Introduction

Electrocardiogram (ECG) remains one of the most important diagnostic tools in the healthcare profession in terms of screening, early diagnosis, and treatment of cardiovascular diseases (CVD) [1]. Accurately interpreting ECG by medical specialists dramatically improves treatment outcomes, especially in acute myocardial infarction or cardiac arrests [2]. Having a direct correlation with patient mortality, a timely approach to ECG can be lifesaving [3]. The true advantage of taking and observing an ECG solely resides in the physician’s competency in accurately interpreting the ECG [3]. As a result, teaching ECG to medical students is one of the most important and challenging syllabi worldwide [4, 5].

A rapid change in medical education practice has begun since 2019 with the emergence of the COVID-19 global pandemic, which disrupted the healthcare system worldwide [6]. Considering the highly contagious nature of the virus, traditional courses could not have continued routinely, and online courses were proposed as an alternate method to teach and provide lectures to medical students [7, 8]. The potential benefits or drawbacks related to the efficacy of the noted structural change in means of education produced conflicted findings. While reporting lower levels of learning, confidence, and engagement compared to the previously employed face-to-face method, the grades remained unchanged or improved significantly [9, 10]. A meta-analysis demonstrated that ECG competence was not different between students regarding face-to-face and online instruction [11]. On the other hand, a recent study showed that using a blended learning model significantly improved learners’ performance and confidence in ECG interpretation [12]. Another study on undergraduate nursing students showed similar results [13].

Considering these results, we were encouraged to conduct a study and compare the effectiveness of online, face-to-face, and blended classes in medical students’ ECG learning.

## Methods

### Study design and population

#### Participants

The study population consisted of medical students and post-graduate junior residents. Two hundred and fifteen medical interns (6th year of medical education) and junior cardiology residents of Shiraz University of Medical Sciences, Shiraz, Iran, participated in this study from February 2021 to February 2022 to compare online, conventional face-to-face, and blended methods.

#### Study design

Participants, regardless of their grades, were divided into three groups. The mode of learning in each group was lecture-based learning. We assumed that all participants, regardless of their grade, had no information regarding ECG interpretation.

The first group was taught ECG for 20 h face-to-face (N = 35), the second group spent 20 h learning online (N = 85), and the third group (N = 95) learned ECG through a blended method which consisted of 10 h face-to-face and 10 h online. For online courses, we used Skype (Skype Technologies, 2021. Skype, Available at: https://www.skype.com), whereas face-to-face courses were held in traditional classrooms. In either method, both students and instructors could ask and answer questions. The online courses were synchronous, and students had to participate twice a week in the online subgroup and once a week in the blended subgroup. Regardless of the subgroups, each course took place in ten two-hour sessions. The same instructors and similar teaching material, including PowerPoint slide sets and media, were used for all participants regardless of their study subgroups.

We applied the convenience sampling method and all interns and residents who had to learn ECG for the first time in their course during the study period entered the study. Since the study was performed in Covid-19 pandemic, the students in each subgroup were selected by their choice.

### Study tool

#### Demographic questions

Before being tested for assessing their ability to ECG interpretation, participants were asked to answer a questionnaire that consisted of Four demographic, including their age, gender, preferred learning method (face-to-face or online classes), and satisfaction level (scored 1 to 5 Likert scale, with 5 showing the highest) regarding face-to-face and online education.

#### Self-assessment questions

The self-assessment questionnaire consisted of nine questions. The participants were asked to score their skills in ECG interpretation regarding determining the patient’s heart rate, heart rhythm, heart axis, ventricular hypertrophy, atrioventricular block (AV-block), bundle branch block (BBB), anatomical location of myocardial infarction (MI), and electrolyte imbalance (scored by 1 to 5 Likert scale, 5 showed the highest skill). For each correct answer, 1 point was given, and a total score between 0 and 9 was calculated for each participant. The higher total score in the self-assessment test before the exam was assumed to indicate participants’ confidence in ECG interpretation.

#### Exam questions

**Fig. 1:**
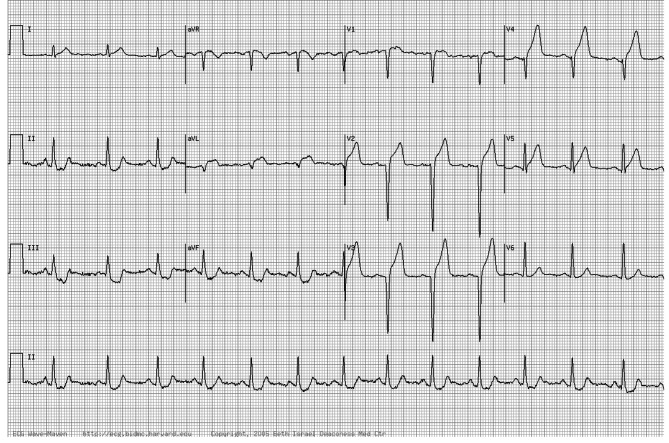
ECG that is used to evaluate students 1. Determine the heart rate. A: 50 B: 80 C: 110 2. Determine the heart rhythm. A: sinus rhythm B: junctional rhythm C: low atrial rhythm 3. Determine the heart axis. A: Right axis B: Left axis C: Normal axis 4. Determine the size of the atria. A: Biatrial normal size B: Left atrium enlargement C: Right atrium enlargement 5. Determine the kind of ventricular hypertrophy. A: left ventricular hypertrophy B: right ventricular hypertrophy C: no ventricular hypertrophy 6. Determine the type of atrioventricular block. A: first-degree AV block B: first-degree Mobitz C: Normal PR interval 7. Determine the type of bundle branch block. A: left BBB B: right BBB C: no BBB 8. Determine the anatomical location of MI. A: anterior MI B: inferior MI C: posterior MI 9. Determine the type of electrolyte imbalance. A: hypocalemia B: hypercalemia C: normal

All participants sat for a 20-minute multiple-choice question exam. Regarding the education they received, nine questions were determined by each section instructor regarding diagnosing heart rate, heart rhythm, heart axis, ventricular hypertrophy, atrioventricular block (AV-block), bundle branch block (BBB), anatomical location of myocardial infarction (MI), and electrolyte imbalance. They were given a copy of an actual patient ECG and asked to answer nine questions. For each correct answer, 1 point was given, and a total score between 0 and 9 was calculated for each participant. Figure 1 shows the ECG and questions.

### Statistical analysis

Descriptive data were presented as mean ± standard deviation (SD), frequency, and percentage. Pearson’s R correlation (*r*), Chi-square test, independent sample t-test, and one-way ANOVA test were used for bivariate analysis. A two-sided p-value (p) of less than 0.05 was considered statistically significant. IBM SPSS Statistics for Windows, version 26.0 (IBM Corporation, Armonk, New York, USA) was used for analysis.

### Ethical consideration

This study was approved by the Ethics Committee of the Shiraz University of Medical Sciences (IR.SUMS.MED.REC.1401.190). The first page of the questionnaire consisted of a consent form that clearly stated that they were free to withdraw at any time without giving a reason. All information they provided would be kept anonymous and confidential. To keep the information anonymous each participant was a given a random code at the beginning of the assessment to write it down on all questionnaires as well as exam paper. The codes were unknown to investigators.

## Results

Two hundred and fifteen participants were studied, including 105 (48.8%) males and 110 (51.2%) females. The mean age of males and females was 25.57 ± 4.48 and 25.51 ± 2.57 years, respectively (p = 0.90). Twenty-six (12.1%) participants were residents, and 189 (87.9%) were interns.

Thirty-five (16.3%), 85 (39.5%), and 95 (44.2%) participants were taught ECG through face-to-face, online, and blended methods, respectively.

Regarding participants’ preferences on teaching methods, 118 (54.9%) preferred face-to-face learning, and the remaining 97 (45.1%) chose online learning (p < 0.001).

Although participants in the blended method seemed more confident about their knowledge in almost half of the questions, their mean total self-assessment score was similar (p = 0.108) (Table 1).

**Table 1:** Association between mean pre-test self-assessment score and teaching methods

Item (Scores up to 5)	Face to face	Online	Blended	p-Value
Q1: Heart Rate	4.31 ± 0.90	4.31 ± 1.12	4.41 ± 0.89	0.789
Q2: Heart Rhythm	3.68 ± 1.13	3.81 ± 1.23	4.04 ± 0.83	0.159
Q3: Heart Axis	3.71 ± 1.40	4.04 ± 1.24	4.35 ± 0.82	**0.010**
Q4: Atrial Size	3.34 ± 1.49	3.60 ± 1.29	3.82 ± 1.09	0.134
Q5: Ventricular Hypertrophy	3.20 ± 1.45	3.36 ± 1.28	3.69 ± 0.96	0.056
Q6: AV-Block	3.42 ± 1.31	3.15 ± 1.26	3.63 ± 1.0	**0.024**
Q7: Bundle-branch Block	3.25 ± 1.24	3.34 ± 1.30	3.85 ± 0.85	**0.002**
Q8: Anatomical location of MI	3.65 ± 1.05	3.54 ± 1.15	3.95 ± 0.79	**0.018**
Q9: Electrolyte imbalance	3.11 ± 1.32	2.88 ± 1.22	3.16 ± 1.07	0.253
Mean total self-assessment score	3.11 ± 1.05	3.11 ± 0.98	3.36 ± 0.65	0.108

AV-Block: Atrioventricular Block, MI: Myocardial infarction

The mean satisfaction score was similar in participants regarding face-to-face (3.30 ± 1.04) and online 3.61 ± 1.08) learning (p = 0.919).

Pre-test self-assessment showed that the mean score for participants’ knowledge of ECG was 3.22 ± 0.86 (0ut of 5). This score had a direct correlation with the mean total test score of 6.55 ± 2.11 (out of 9) (*r* = 0.355, p < 0.001).

The blended method seemed more promising in almost half of the exam questions regarding teaching method effectiveness. The mean exam total score was also significantly higher in participants who were taught blended than in the others (p = 0.017) (Table 2). Face-to-face and online teaching methods had similar efficacy regarding mean total score (p = 0.819). The residents (6.5 ± 1.83) and interns (6.5 ± 2.15) had similar mean total scores (p = 0.891).

**Table 2:** Association between correct answers and teaching methods

Item	Face-to-face (N = 35)	Online (N = 85)	Blended (N = 95)	p-Value
Q1: Heart Rate	28 (80%)	72 (84.7%)	84 (88.4%)	0.459
Q2: Heart Rhythm	29 (82.9%)	71 (83.5%)	90 (94.7%)	**0.035**
Q3: Heart Axis	27 (77.1%)	71 (83.5%)	87 (91.6%)	0.075
Q4: Atrial Size	15 (42.9%)	39 (45.9)	69 (72.6%)	**< 0.001**
Q5: Ventricular Hypertrophy	14 (40%)	41 (48.2%)	50 (52.6%)	0.437
Q6: AV-Block	28 (80%)	59 (69.4%)	83 (87.4%)	**0.013**
Q7: Bundle-branch Block	26 (74.3%)	55 (64.7%)	75 (78.9%)	0.099
Q8: Anatomical location of MI	27 (77.1%)	66 (77.6%)	86 (90.5%)	**0.04**
Q9: Electrolyte imbalance	15 (42.9%)	42 (49.4%)	60 (63.2%)	0.059
Mean total score (out of 9)	5.97 ± 2.33	6.07 ± 2.07	7.20 ± 1.89	**0.017**

AV-Block: Atrioventricular Block, MI: Myocardial infarction

## Discussion

In this study, we found that the blended method was a better learning approach that led to higher participant scores. However, it showed that most participants preferred face-to-face courses to online ones.

Our study showed that the mean total score of students who learned ECG using a blended learning approach was significantly higher than other groups. At the same time, face-to-face and online methods had similar efficacy. Similar to our result, Viljoen et al. showed that a blended learning method leads to better ECG learning in medical students than face-to-face and online methods [14]. Another study by Liu et al. reported that blended teaching is more effective than traditional face-to-face and online methods [15]. It was also demonstrated that the benefit of adding online learning to convectional face-to-face lectures was greatest when students had unlimited access to computer-assisted ECG training [11]. Blended learning may lead to better ECG comprehension for students for various reasons. Blended learning can provide students with increasing the net amount of time they spend on ECG learning rather than wasting their time in long time-consuming face-to-face courses and the ability to contract with each other and their instructors to ask questions and solve their problems.

Regarding students’ preferences, our study showed that most of them preferred face-to-face courses to online ones. Similar to this result, Nepal et al. reported that medical students found traditional face-to-face classes were more effective than online courses [16]. Also, Saurabh et al. found that face-to-face learning was preferred among medical students to online learning [17]. In contrast to these findings, participants in Rastogi et al.‘s research preferred the online learning method, which was also confirmed by Sandhaus et al.‘s study [18, 19]. These differences in perspectives can be clarified by the quantity and quality of interaction, content quality, internet connectivity, and trainee and trainer’s digital literacy.

With social distancing protocols mandated by the COVID-19 pandemic, online learning has become the predominant approach to keeping up with medical education [20]. Studies have supported the importance of social presence and interaction in learning, which can be difficult to achieve online. Online learning presents several challenges, such as limited interaction and discussion among students, difficult communication with instructors, and restricted time for query resolution [21, 22]. Also, According to previous studies, participants have reported privacy issues and technical challenges, such as unstable internet connections associated with online learning [23–25]. Despite the presumed difficulties with online teaching, the distancing protocols to prevent COVID-19 infection mandated us to move from conventional face-to-face teaching to online methods, like other medical schools worldwide.

### Limitations and strengths

The current research shares the fundamental issue of self-reported surveys with other internet surveys. The study’s fundamental nature, such as its sample method, might have led to selection bias as it is only exposed to those with internet access and those who speak the Persian language. Another limitation of the study was the need for pre-test self-assessment. We assumed that all participants, regardless of their grades, had no information regarding ECG interpretation. It would be better to consider that in future studies. Also, The assessment tool for evaluating participants’ skill to interpret ECG was not a standard validated questionnaire. To the best of our knowledge, the current research is one of the pioneer investigations to examine medical interns’ and residents’ perspectives, preferences, satisfaction levels, and suggestions for both learning approaches. Given the suddenness of the Covid-19 outbreak and the timing of the events, further studies are needed to properly understand the impact of the radical change in teaching strategies on medical students and the results regarding their comprehensive and interpretive abilities.

## Conclusion

In conclusion, while most students preferred face-to-face learning to online learning, a blended method seemed more promising regarding students’ skill enhancement to interpret ECG. The results of this study implicate that it would be better for medical students and medical school faculties to move from conventional face-to-face learning approaches to more modern approaches like blended ones. However, we recommend further studies with different topics and more extensive study groups.

## Data Availability

SPSS data of the participants can be requested from the authors. Please write to the corresponding author if you are interested in such data.
